# New Method of Applying Collodion for Producing Occlusion of the Eyelids

**Published:** 1851-11

**Authors:** 


*New Method of Applying Collodion for producing Occlusion of the Eyelids.*—The process of occlusion of the eyelids, by bringing them together and applying collodion over them, presents two inconveniences. If a drop of the collodion reaches the conjunctiva, it produces irritation always very painful, and especially troublesome if, as is generally the case, the membrane is already inflamed. In the second place, if it be desired to examine the eye before the collodion has spontaneously detached itself, it becomes necessary to remove it with ether or scissors—both delicate and irritating processes, often causing pain and apprehension to the patients.

M. Barrier, of Lyons, employs a process which avoids these difficulties. A thread is placed vertically on the upper eyelid; a small piece of fine linen is then dipped in collodion, and placed on the lid, a little above the root of the eyelashes, so as to fasten down the thread. The same thing is done on the lower lid. To keep the lids closed, it is only necessary to tie the threads in a knot or bow; and it is quite easy to open or separate the eyelids.—*Annales d' Oculistiques*, as quoted in *Gazette Médicale*, August 16, 1851.

